# Independent associations of mental health and diabetes complications with health‐related quality of life: Evidence from a cross‐sectional study

**DOI:** 10.1111/dom.70434

**Published:** 2026-01-07

**Authors:** Norbert Hermanns, Philip Kittel, Paco Cerletti, Bernhard Kulzer, Dominic Ehrmann

**Affiliations:** ^1^ Research Institute Diabetes Academy Mergentheim (FIDAM) Bad Mergentheim Germany; ^2^ Department of Clinical Psychology and Psychotherapy University of Bamberg Bamberg Germany; ^3^ diateam Bad Mergentheim Germany; ^4^ Roche Diagnostics International Ltd. Rotkreuz Switzerland; ^5^ Roche Diagnostics International AG Basel Switzerland

**Keywords:** health economics, observational study, patient reported outcomes, real‐world evidence

## Abstract

**Aims:**

Health‐related quality of life (HRQoL) is a key patient‐reported outcome in diabetes care, yet the extent to which somatic and psychological factors are associated with HRQoL remains unclear. This study examined how demographic, diabetes‐related, medical, and psychological factors were independently associated with HRQoL in adults with diabetes.

**Materials and Methods:**

A cross‐sectional online survey was conducted among adults with diabetes in Germany (September 2024–February 2025). HRQoL was assessed using the EuroQol 5‐Dimension 5‐Level questionnaire (EQ‐5D‐5L). Participants also completed the PHQ‐8 for depressive symptoms, the problem areas in diabetes (PAID) scale, and the hypoglycaemia fear survey (HFS‐II). Clinical variables were self‐reported and included diabetes type, duration, HbA1c, body mass index (BMI), and complications. Tobit regression accounted for the censored EQ‐5D distribution. Blockwise multivariable models evaluated incremental explained variance across demographic, diabetes‐related, comorbidity, and mental‐health domains.

**Results:**

Of 1581 invitees, 734 completed the EQ‐5D (mean age 56 ± 14 years; 73% type I). In multivariable analyses, female sex (β = −0.045), higher BMI (β = −0.029), diabetic foot syndrome (β = −0.078), neuropathy (β = −0.123), and elevated depressive symptoms (β = −0.212), diabetes distress (β = −0.069), and fear of hypoglycaemia (β = −0.085) were all independently associated with lower EQ‐5D utilities (*p* < 0.01). Mental‐health variables explained a similar proportion of variance (≈22%) as diabetes‐related complications (≈20%). Mental health factors like depression, diabetes distress, and fear of hypoglycaemia showed highly significant associations with reduced HRQoL by up to 27%.

**Conclusions:**

Both diabetes complications and mental health determine HRQoL in people with diabetes. Depression emerged as the strongest independent predictor reducing HRQoL by up to 21%. This underscores the importance of mental health for HRQoL. This findings highlight the relevance of integrating mental health assessment into diabetes management.

## 1 INTRODUCTION

Diabetes mellitus is associated with a substantial burden for people with diabetes. Diabetes‐related acute or long‐term complications, concomitant diseases as well as distress associated with its treatment and self‐management have a negative impact on health‐related quality of life (HRQoL).^1^ HRQoL has received particular attention, as it reflects how diabetes, its complications and its treatment affect daily life. For the evaluation of diabetes care respectively new pharmaceutical or technological intervention it is also of interest to which extent HRQoL can be improved beyond biomedical indicators.^1^, ^2^, ^3^, ^4^, ^5^ Widely used instruments in both research and clinical care are short‐form health survey (SF‐36)^6^ or the EuroQol‐5D (EQ‐5D),^7^, ^8^ which assess self‐reported physical, social and emotional functioning. In addition, these HRQoL scores can be converted to utility scores, which can be incorporated into health economic evaluations to quantify the effect of an intervention on HRQoL, providing valuable information for informing healthcare decisions.

For clinical care aiming to optimise quality of life in people with diabetes, it is important to understand which factors are associated with reduced quality of life. It is well known that, for example, severe complications in diabetes are associated with lower HRQoL,^9^, ^10^, ^11^, ^12^ or that female gender, shorter diabetes duration and younger age are more often linked to higher perceived diabetes burden.^13^, ^14^ In addition, recent evidence suggests that mental health factors play an important role and are strongly associated with HRQoL, as a longitudinal study demonstrated that changes in depression and anxiety scores over 3 months were strong predictors of changes in EQ‐5D scores (utility) values.^15^, ^16^ Given the broadness of demographic, medical and mental health related associates with HRQoL, there is currently a gap in understanding the relative importance of these associates for HRQoL. For example, older age, diabetes complications, and psychological distress may each affect HRQoL, but their effects frequently overlap. To clarify these relationships, it is important to distinguish univariate from multivariate associations to examine how demographic, medical, and mental health factors are independently associated with HRQoL. Understanding which factors are independently associated with HRQoL may support the identification of subgroups at increased risk for impaired HRQoL in clinical practice. In addition, demographic, medical, or mental health factors with stronger independent links to lower HRQoL can inform the prioritisation of potential intervention targets.^16^, ^17^, ^18^

Therefore, a cross‐sectional study in Germany was performed allowing the comparison of univariate and multivariate associations between demographic, medical and health‐related variables and HRQoL. This allows for identifying factors that are independently associated with HRQoL beyond shared variance or confounding. The present study therefore aimed to identify independent associations between HRQoL and demographic, diabetes‐related, medical, and mental health factors with HRQoL among adults with diabetes in Germany.

## 2 METHODS

### 2.1 Design

People with diabetes aged 18 years or older were invited to participate in a cross‐sectional online survey in Germany. Participants were recruited from the German diabetes panel dia•link (www.dialink-diabetes.de), an online panel where people with diabetes can register free of charge to take part in regular surveys on diabetes‐specific issues. Members of the dia•link panel with diabetes received a notification via the panel website and were fully informed about the survey. An e‐mail invitation was sent to 1581 dia•link members with diabetes who could access the survey between September 20th 2024 and February 28th 2025. In addition, participants were invited at a “T1Day for people with type 1 diabetes” on 26th January 2025 and in an inpatient setting at the Diabetes Center Bad Mergentheim, Germany, who were provided with the same link as the panel members (see Figure S1). All participants provided informed consent for study participation before the start of the survey. This study received ethical approval from the competent ethics committee (HermannsNorbert‐12‐18VA).

### 2.2 Outcome measures

For this analysis, the following variables were assessed by self‐report of the study participants: demographic variables: gender, age, migration background, diabetes‐specific variables: diabetes duration, body mass index (BMI), HbA1c, presence of diabetic foot syndrome, diabetic neuropathy, retinopathy, nephropathy, amputation, history of severe hypoglycaemia or diabetic ketoacidosis; other medical conditions: cancer, cardiovascular disease, lung disease or stroke and psychological outcomes. In addition participants also completed the German versions of the PhQ‐8 Depression questionnaire for assessment of depressive symptoms^19^ of the problem areas in diabetes (PAID) questionnaire for diabetes‐related distress^20^ and Fear of hypoglycaemia worry scale II.^21^ For the measurement of HRQoL we used the EuroQol 5‐Dimension 5‐Level questionnaire (EQ‐5D‐5L) with German normative data.^22^, ^23^

### 2.3 Statistical analysis

To examine the influence of sociodemographic, clinical, and psychosocial characteristics on HRQoL, Tobit regression models were employed using the censReg package in R. The EQ‐5D‐5L utility scores as the dependent variable exhibited a censored distribution, with a lower bound at −0.2 and an upper bound at 1.0, as defined by the distribution of the utility scores and German EQ‐5D‐5L value set. Initially, univariate analyses were performed with EQ‐5D‐5L utility as the dependent variable and demographic, diabetes‐related, medical, and psychological variables as predictors. This step allowed us to identify variables with significant bivariate associations with EQ‐5D‐5L and to reduce the number of candidate predictors for subsequent multivariable modelling. In the univariate analyses, all predictors with a *p*‐value <0.15 were considered eligible for inclusion in the subsequent multivariate models.

Age, diabetes duration, BMI and HbA1c were z‐standardised to ensure comparability of regression coefficients across variables measured on different scales. Coefficients of z‐standardised values indicate utility changes per SD. Both the presence of a specific complication or the presence of elevated depression scores (PHQ‐8 ≥ 10),^24^ elevated diabetes distress (PAID ≥40)^25^ or elevated fears of hypoglycaemia (HFS II >36)^26^ were dichotomised and analysed as a binary variable (yes vs. no). The regression coefficients indicate the change of the utility score if a condition was present compared to the reference group without this specific condition respectively.

A stepwise and block‐wise multi‐variate Tobit regression analysis with the remaining variables was performed in order to identify independent associations with EQ‐5D‐5L utility. Multivariate modelling controls for potential confounding and allows estimation of the unique association of each predictor. The block‐wise approach further enabled us to assess the incremental explanatory power of different groups of variables (demographic, diabetes‐related, comorbidities, psychological factors). Model selection within each block was based on Akaike's Information Criterion (AIC) using a stepwise forward–backward procedure.

To evaluate model fit, we calculated for each block McKelvey and Zavoina's pseudo‐R^2^ (R^2^‐MZ), which has been recommended for censored dependent variables. R^2^‐MZ is conceptually closest to the coefficient of determination (R^2^) in linear regression. It can be interpreted as the proportion of variance in the underlying latent outcome that is explained by the associated factors.

In addition, we performed a robustness analysis in which we repeated the stepwise multivariate analysis with the EQ‐5D utility index, this time corrected for the depression/anxiety dimension to investigate to which extent the substantial association with depressive symptoms was influenced by the depression/anxiety dimension of the EQ‐5D utility score.

A second robustness check was performed using multivariable Tobit models stratified by diabetes type, sex, and recruitment setting to verify that observed associations were consistent across key subgroups and not driven by sample composition. Omnibus interaction tests were used, in addition to stratified analyses, to formally test whether associations differed across the specified subgroups. All models were estimated using the maximum number of available cases for the respective set of predictors.

## 3 RESULTS

Of the 1581 persons who were contacted to participate in the study, 734 persons with diabetes completed the EQ‐5D questionnaire. Most participants had type I diabetes (73%) and were slightly more often female (54%). Mean age was 56.3 years and only 9% of the sample reported a migration background. Diabetic neuropathy was the most common microangiopathic complication followed by retinopathy. Cardiovascular disease was the most common macroangiopathic complication, whereas acute complications such as severe hypoglycaemia (requiring third party assistance for recovery) or diabetic ketoacidosis were rare. In summary, the study participants reported a mean number of additional complications of 0.98 and an average EQ‐5D‐5L‐utility score of 0.85. See Table 1 for more details of the sample description.

**TABLE 1:** Sample description.

Characteristics	Mean ± SD; number/total	*N* responses resp. %
Age (years)	56.32 ± 14.529	*N* = 738
Sex		
Male	342/738	46.3%
Female	396/738	53.7%
Migration background		
No	653/721	90.6%
Yes	68/721	9.4%
Diabetes duration (years)	25.11 ± 16.766	*N* = 700
BMI	27.65 ± 5.96	*N* = 742
HbA1c (%)	6.75 ± 1.04	*N* = 617
Diabetes type		
Type 1 diabetes	540/740	73.0%
Type 2 diabetes	167/740	22.6%
Other type	33/740	4.5%
Severe hypoglycaemia		
No	695/743	93.5%
Yes	48/743	6.5%
Diabetic ketoacidosis		
No	717/730	98.2%
Yes	13/730	1.8%
Retinopathy		
No	574/717	80.1%
Yes	143/717	19.9%
Neuropathy		
No	548/726	75.5%
Yes	178/726	24.5%
Amputation		
No	703/712	98.7%
Yes	9/712	1.3%
Cancer		
No	667/714	93.4%
Yes	47/714	6.6%
Cardiovascular disease		
No	621/718	86.5%
Yes	97/718	13.5%
Stroke		
No	694/715	97.1%
Yes	21/715	2.9%
Lung disease		
No	671/709	94.6%
Yes	38/709	5.4%
Average number of complications	0.98 ± 1,32	743
Diabetes distress (PAID)	27.38 ± 21.24	770
Fear of hypoglycaemia (HFS_worry)	17.12 ± 14.37	741
Depressive symptoms (PHQ_8)	6.36 ± 5.13	722
Health‐related quality of life (EQ‐5D‐5L)	0.85 ± 0.20	734

The results of the univariate Tobit regression analysis are described in Table 2. Female sex, having not type 1 diabetes, higher BMI, elevated HbA1c, occurrence of severe hypoglycaemia, diabetic foot syndrome (DFS), nephropathy, neuropathy, amputation, cardiovascular disease, stroke, lung disease, as well as psychological outcomes like elevated hypoglycaemia fear, depression scores or Diabetes Distress scores were associated with lower utility scores. The two strongest associations to HRQoL were the presence of a DFS and diabetic neuropathy (see further details in Table 2).

**TABLE 2:** Results of univariate associations between EQ‐5D utility index and demographic, diabetes‐related, medical and psychosocial variables (results of univariate Tobit regression analyses).

Variable	Contrast	Estimate	CI	*p*‐Value
Demographic variables				
Age	Per 1‐SD increase	−0.014	−0.032 to 0.004	0.122
Sex	Sex: female vs. male	−0.041	−0.077 to −0.005	0.026
Migration background	Migration background: yes vs. no	0.041	−0.021 to 0.102	0.197
Diabetes‐related variables				
Diabetes duration	Per 1‐SD increase	−0.001	−0.019 to 0.017	0.917
BMI	Per 1‐SD increase	−0.057	−0.074 to −0.040	<0.001
HbA1c	Per 1‐SD increase	−0.035	−0.054 to −0.016	<0.001
Diabetes type	Diabetes type: type 2 vs. type 1	−0.095	−0.137 to −0.052	<0.001
Diabetes type	Diabetes type: other type vs. type 1	−0.075	−0.161 to 0.011	0.087
Severe hypoglycaemia	Severe hypoglycaemia: yes vs. no	−0.096	−0.167 to −0.024	0.009
Diabetic ketoacidosis	Diabetic ketoacidosis: yes vs. no	−0.030	−0.163 to 0.103	0.657
Diabetic foot syndrome	Diabetic foot syndrome: yes vs. no	−0.212	−0.262 to −0.161	<0.001
Retinopathy	Retinopathy: yes vs. no	−0.043	−0.088 to 0.002	0.064
Nephropathy	Nephropathy: yes vs. no	−0.176	−0.247 to −0.105	<0.001
Neuropathy	Neuropathy: yes vs. no	−0.201	−0.240 to −0.162	<0.001
Amputation	Amputation: yes vs. no	−0.101	−0.260 to 0.059	0.217
Other medical conditions				
Cancer	Cancer: yes vs. no	−0.039	−0.112 to 0.034	0.300
Cardiovascular disease	Cardiovascular disease: yes vs. no	−0.102	−0.154 to −0.050	<0.001
Stroke	Stroke: yes vs. no	−0.137	−0.245 to −0.029	0.013
Lung disease	Lung disease: yes vs. no	−0.132	−0.212 to −0.052	0.001
Psychological variables				
Diabetes distress	PAID score elevated: yes vs. no	−0.211	−0.248 to −0.175	<0.001
Depressive symptoms	PHQ score: yes vs. no	−0.273	−0.308 to −0.238	<0.001
Hypoglycaemia worries	HFS II score: yes vs. no.	−0.209	−0.260 to −0.158	<0.001

**FIGURE 1:**
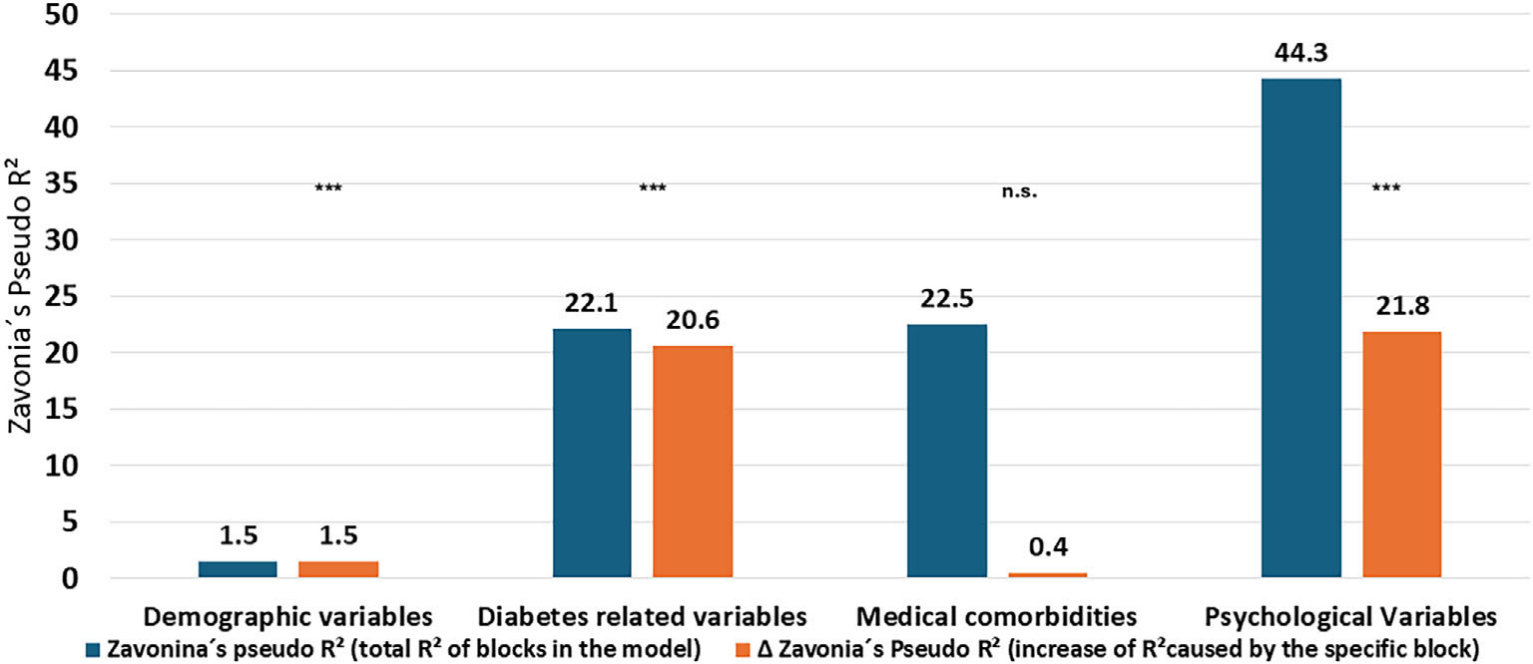
Model fit expressed as Zanovia's pseudo R^2^ of the four blocks of the multivariate Regression model.

**FIGURE 2:**
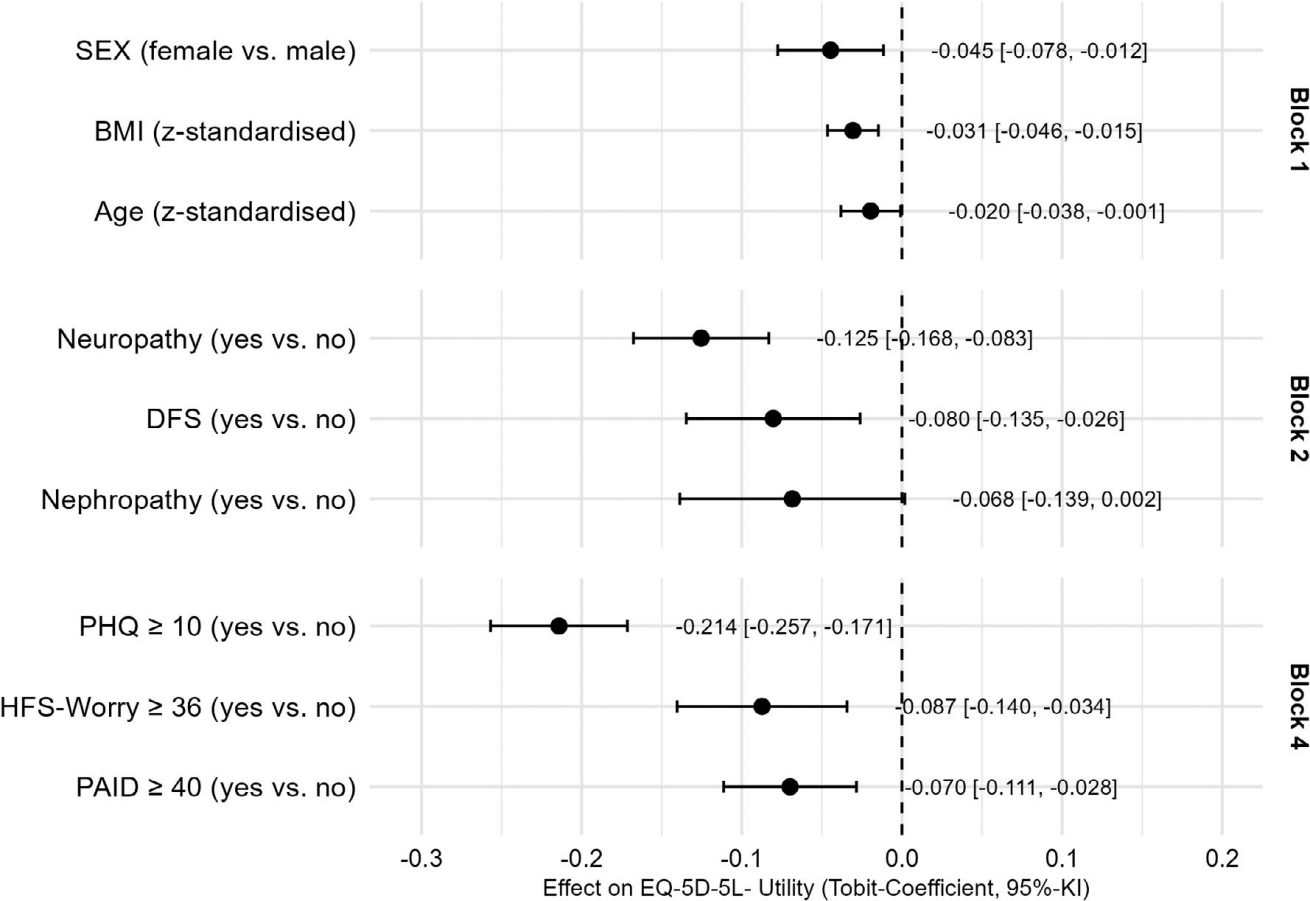
Results of the stepwise multivariate Tobit regression analysis between EQ‐5D_L5_utility_index (dependent variable) and potential statistical predictors (independent variable) with *p*‐values <0.15 in univariate analysis (note block 3 was deleted in the stepwise analysis due to the low contribution to explained variance of block 3).

Using step‐ and block‐wise Tobit regression, we identified the factors that were independently associated with HRQoL (see Figure 1). Demographic variables explained 1% of the variance. Diabetes‐related factors significantly increased the explained variance by about 20.6%, whereas other factors and comorbidities added little beyond these (+0.29%). Notably, psychological variables contributed a further 21.8% to the explained variance (see Figure S2 for the complete Tobit regression model). In the demographic block, sex emerged as a significant associated factor, with female participants showing HRQoL scores that were 4.5% lower on the utility scale (β = −0.045) compared to male participants. The block consisting of diabetes related variables increase of BMI by one standard deviation (6.0 kg/m^2^) reduced the utility index by 3.1%. The presence of diabetic foot syndrome and diabetic neuropathy reduced HRQoL by 8.0% respectively 12.5%. Because the other medical conditions in block 3 provided no additional explanatory value, the AIC‐based selection process excluded this block from the final model shown in Figure 2. The last block with psychological variables showed that the elevated PHQ8‐depression scores (−21.4), diabetes distress scores (7.0%) and fear of hypoglycaemic (8.7%) were also remaining significantly associated with the utility index in the fourth block (see Figure 2).

**FIGURE 3:**
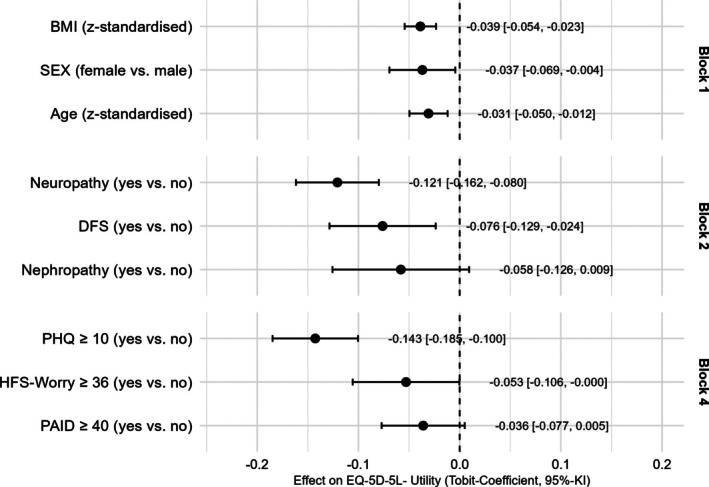
Results of robustness analysis; stepwise multivariate Tobit regression analysis between EQ_5D_5L_utility_index without the depression/anxiety dimension (dependent variable) and potential statistical predictors (independent variable) with *p*‐values <0.15 in univariate analysis (note block 3 was deleted in the stepwise analysis due to the low contribution to explained variance of block 3).

To test robustness, we conducted a sensitivity analysis using the utility index excluding the anxiety/depression dimension (Figure 3). The blockwise McKelvey and Zavoina's pseudo‐R^2^ results were comparable to the main analysis: demographics explained 2.7% of the variance; adding diabetes‐related variables increased explained variance by 22.4%; adding other medical conditions raised it slightly by 0.5%; and adding psychological variables further increased it by 10.8 to a total variance explanation of 36.4%. Overall, this was ~10% lower than in the original model. Each block significantly improved model fit, except the addition of other medical conditions. The diabetes‐related comorbidities of the main analysis remained significant. However, the associations between HRQoL and elevated diabetes distress and fear of hypoglycaemia became lower and no longer statistically robust (PAID ≥40: β = −0.036, *p* = 0.1092; HFS ≥36: β = −0.053, *p* = 0.0510), whereas PHQ ≥10 remained independently associated with lower utility (β = −0.143, *p* < 0.0001). This pattern may indicate that the effects of diabetes‐related distress and fear of hypoglycaemia on EQ‐5D are largely mediated through the psychological dimension (see also Figure S2 for the complete Tobit regression model without utility dimension of depression/anxiety).

A second robustness check was performed using multivariable Tobit models stratified by diabetes type, gender and recruitment setting. Estimates closely mirrored the main analysis in direction and magnitude (e.g., PHQ ≥10: T1D β ≈ −0.19; T2D β ≈ −0.26; see Figures S3–S5). Omnibus interaction tests indicated no heterogeneity by diabetes type or recruitment; a small, nominal signal for sex (*p* ≈ 0.025) did not alter conclusions (Table S1). Overall, associations were consistent across strata, supporting the robustness and generalisability of the primary findings (Table S2).

## 4 DISCUSSION

In this study, we examined a broad range of demographic, clinical, and psychosocial factors to identify independent associations of HRQoL among people with diabetes in Germany. The mean utility score of our sample (0.85) matches well to the expected utility score of a representative German sample with 4998 participants, which was also 0.85 for people with two medical conditions,^23^ which is in this sample diabetes and one additional comorbity. In univariate analysis, diabetic foot syndrome, neuropathy, nephropathy, stroke and lung disease showed the most important somatic associations for lower HRQoL with a magnitude of −21% to −13% of the total scale range. There was also a significant impact of severe hypoglycaemia on HRQoL (−9.6%), whereas diabetic ketoacidosis was not associated with lower HRQoL. Compared to these acute complications psychological factors like depression, diabetes distress and fear of hypoglycaemia showed also highly significant associations with reduced HRQoL by up to 21%.

These results align with international evidence showing that somatic complications substantially affect quality of life.^10^, ^15^ Previous studies have also demonstrated that depressive symptoms are among the strongest predictors of impaired HRQoL in diabetes.^27^, ^28^ Similarly, diabetes distress and fear of hypoglycaemia have increasingly been recognised as important factors associated with daily functioning and well‐being,^29^, ^30^ and their strong association with HRQoL in our data underlines the clinical relevance of addressing emotional burden in diabetes care. Overall, the univariate results suggest that HRQoL is shaped by both somatic complications and impaired mental health.

In our data, the multivariate models indicated that, in addition to elevated depressive symptoms and diabetes distress, fear of hypoglycaemia was independently associated with HRQoL. The impact of depressive symptoms exceeded those of biomedical complications, and this association remained robust when the depression/anxiety dimension of the EQ‐5D was excluded. The independent association of neuropathy^10^ and diabetic foot syndrome^31^ with lower HRQoL reinforces prior evidence that complications compromising mobility and daily functioning have a particularly strong association with lower quality of life.^15^ The relative lack of explanatory power of other comorbidities such as cardiovascular disease or cancer may reflect the fact that their impact may be mediated through functional impairments already captured by diabetes‐related complications, or that our sample size was insufficient to detect smaller effects.

Given that approximately one third of people with diabetes are affected by elevated depressive symptoms,^32^ elevated diabetes distress^33^ or fear of hypoglycaemia^34^ the finding that these conditions are significantly associated with reduced HRQoL have implications for research, the evaluation of diabetes interventions such as medical devices as well as clinical care. While prevention and treatment of somatic complications remain central goals of diabetes care, addressing issues such as elevated depressive symptoms, diabetes distress, and fear of hypoglycaemia may be associated with an increase in HRQoL of a similar magnitude. These mental health conditions may be candidates for mapping HRQoL in people with diabetes, as it was successfully demonstrated for fear of hypoglycaemia.^35^

Especially elevated scores on these mental health conditions may be amplifiers for the perception of the lower HRQoL; thus, improvements or deterioration of HRQoL may not solely reflect the respective changes of somatic conditions but also changes in mental health variables like depression, diabetes distress or fear of hypoglycaemia.

Clinically, this underscores the need to systematically address mental health in diabetes care through regular monitoring of well‐being at regular visits and when glycaemic control or life circumstances change (e.g., PHQ‐2, PAID‐5,^36^ WHO‐5^35^). A discussion of results with the person is important as elevated scores should be treated as indicators, not as diagnoses. More severe mental health problems (e.g., clinical depression) should be confirmed with clinical interview. Through shared decision‐making, the next steps should be agreed: e.g., targeted diabetes education to close knowledge gaps or enhance skills,^35^, ^37^ peer/group programs for social support,^38^ digital self‐help tools,^39^, ^40^, ^41^ or referral to psychotherapy or psychiatric co‐management^42^ when mental health issues emerge. A reduction of treatment burden and hypoglycaemia risk by using diabetes technology^41^, ^43^ might also be beneficial. Together, these steps offer a practical and scalable framework for embedding psychosocial care into routine diabetes management.

For research it may be helpful to better understand the association between depression and somatic complications and to analyse if all depressive symptoms are associated equally to the diminishing effect on HRQoL.^44^

An additional approach that could ease the mental health burden and empower people with diabetes to feel more in control of their diabetes management would be a higher emphasis on the experience of people with diabetes in health technology assessment.^12^ Medical devices, such as continuous glucose monitoring devices, automated insulin delivery systems, but also digital medical devices, hold the promise of reducing the fear of hypoglycaemia and diabetes distress.^43^, ^45^ By actively including those measurements in health technology assessments, payers and HTA bodies could facilitate access to technological solutions which benefit their lived experience.

This study has several strengths, including a relatively large sample, systematic examination of a wide range of potential associations to HRQoL, and the use of a robust statistical approach tailored to the censored distribution of EQ‐5D utility scores. The sensitivity analysis excluding the depression/anxiety dimension further strengthens the validity of our conclusions, as it rules out the possibility that the association between depressive symptoms and HRQoL is merely an artefact of measurement overlap. In addition, the HRQoL levels in our sample were highly comparable to those reported in a large, representative German dataset,^23^ particularly for individuals with diabetes and one additional comorbidity. Moreover, HRQoL scores did not differ significantly across recruitment sources (panel participants, event visitors, clinical sample), suggesting that our sample is broadly comparable to the wider population of people with diabetes in Germany. These findings support the external validity of our study.

Nevertheless, some limitations should be considered. First, the cross‐sectional design precludes conclusions about causal directions. While it is plausible that depressive symptoms are negatively associated with HRQoL, the reverse—impaired HRQoL might also be associated with depression—may also be true. Longitudinal studies are required to disentangle these bidirectional relationships. A second key limitation is that all variables—including concurrent symptom status—are self‐reported and therefore vulnerable to recall bias. The severity of comorbidities was also not assessed. Future studies should complement self‐report measures with medical records or objective clinical indicators to enhance accuracy and reduce potential reporting bias. Third, while our sample was diverse, recruitment from an online panel and a diabetes clinic may limit generalisability, particularly for individuals with lower digital literacy or reduced healthcare access. In addition, people with type 2 diabetes were underrepresented in our sample. This imbalance, together with the online recruitment strategy, limits the generalisability of our findings. Some comorbid conditions were relatively infrequent in our sample, limiting statistical power to detect their effects. Finally, given the more exploratory character of this study, there was no adjustment of the α‐level to avoid an inflation of the ß‐error.

Future research should validate these results in more representative samples that include a higher proportion of people with type 2 diabetes and use more varied recruitment methods, and ideally replicate them in longitudinal cohorts combining self‐reported outcomes with objective clinical data. Moreover, as mental health factors have emerged as major drivers of diabetes‐specific symptoms,^39^ interventional studies are needed to determine whether effective treatment of mental health problems in people with diabetes leads to measurable improvements in HRQoL and other person‐reported outcomes. Health economic evaluations incorporating these effects could further inform policy decisions on the adoption and implementation of novel treatments and technologies as well as integrated care models.

In conclusion, in this study population demographic variables contributed little to HRQoL, whereas diabetes‐related complications, along with elevated depressive symptoms, diabetes distress, and fear of hypoglycaemia, emerge as important and independent predictors of HRQoL in people with diabetes. Interestingly, the mental health variables identified were as important in predicting HRQoL as the somatic complications of diabetes. These findings underscore the need for stronger integration of mental health care into routine diabetes management and emphasise that improving HRQoL requires addressing both the physical and psychological dimensions of living with diabetes.

## AUTHOR CONTRIBUTIONS

All authors contributed significantly to the manuscript. Norbert Hermanns, Philip Kittel, Paco Cerletti, Bernhard Kulzer and Dominic Ehrmann designed the study. Norbert Hermanns and Dominic Ehrmann collected and analysed the data. Norbert Hermanns wrote the manuscript. Philip Kittel, Paco Cerletti, Bernhard Kulzer and Dominic Ehrmann contributed to the interpretation of the data and revised the manuscript for important intellectual content.

## CONFLICT OF INTEREST STATEMENT

Norbert Hermanns reports Advisory Board member fees from Abbott Diabetes Care and Dexcom as honoraria for lectures from Abbott Diabetes Care. Philip Kittel is an employee of Roche Diagnostics. Paco Cerletti is an employee of Roche Diagnostics. Bernhard Kulzer reports fees as an advisory board member of Abbott Diabetes Care, Embecta, Roche Diabetes Care, Novo Nordisk, Berlin Chemie AG, and Dexcom Germany, as well as fees for lectures from Sanofi Germany, Novo Nordisk, Abbott Diabetes Care, Roche Diabetes Care, Berlin Chemie AG, Embecta, Dexcom, and Feen. In addition, he reports on travel expenses and fees for scientific conferences from Sanofi, Roche Diabetes Care, and Berlin Chemie AG, as well as unpaid commitments as a workshop leader and member of working groups of the German Diabetes Society. Dominic Ehrmann reports Advisory Board member fees from Dexcom Germany and Roche Diabetes Care as well as honoraria for lectures from Abbott Diabetes Care, Sanofi‐Aventis, Dexcom Germany, Boehringer‐Ingelheim/Eli Lilly, Eli Lilly, and Roche Diabetes Care.

## Supporting information


**Data S1:** Supporting information.

## Data Availability

The following data can be shared: individual participant data underlying the results reported in this article after de‐identification (text, tables, figures, and appendices). Additionally, the study protocol can be made available. Data sharing can commence immediately following publication and continue until 10 years of publication. The data will be shared with researchers who provide a methodologically sound proposal. The sharing of the data needs to fulfil the purpose of achieving the aims of the approved proposal. Proposals should be directed to hermanns@fidam.de. To gain access to the data, the requestors will need to sign a data access agreement.
