# Supplementary material for: Independent associations of mental health and diabetes complications with health‐related quality of life: Evidence from a cross‐sectional study
Source: Diabetes Obes Metab. 2026 Jan 7;28(3):2383–92. doi: 10.1111/dom.70434 (PMC12890743; doi:10.1111/dom.70434)
Supplement: Supplementary file 1 — Data S1: Supporting information. [file DOM-28-2383-s001.pdf]

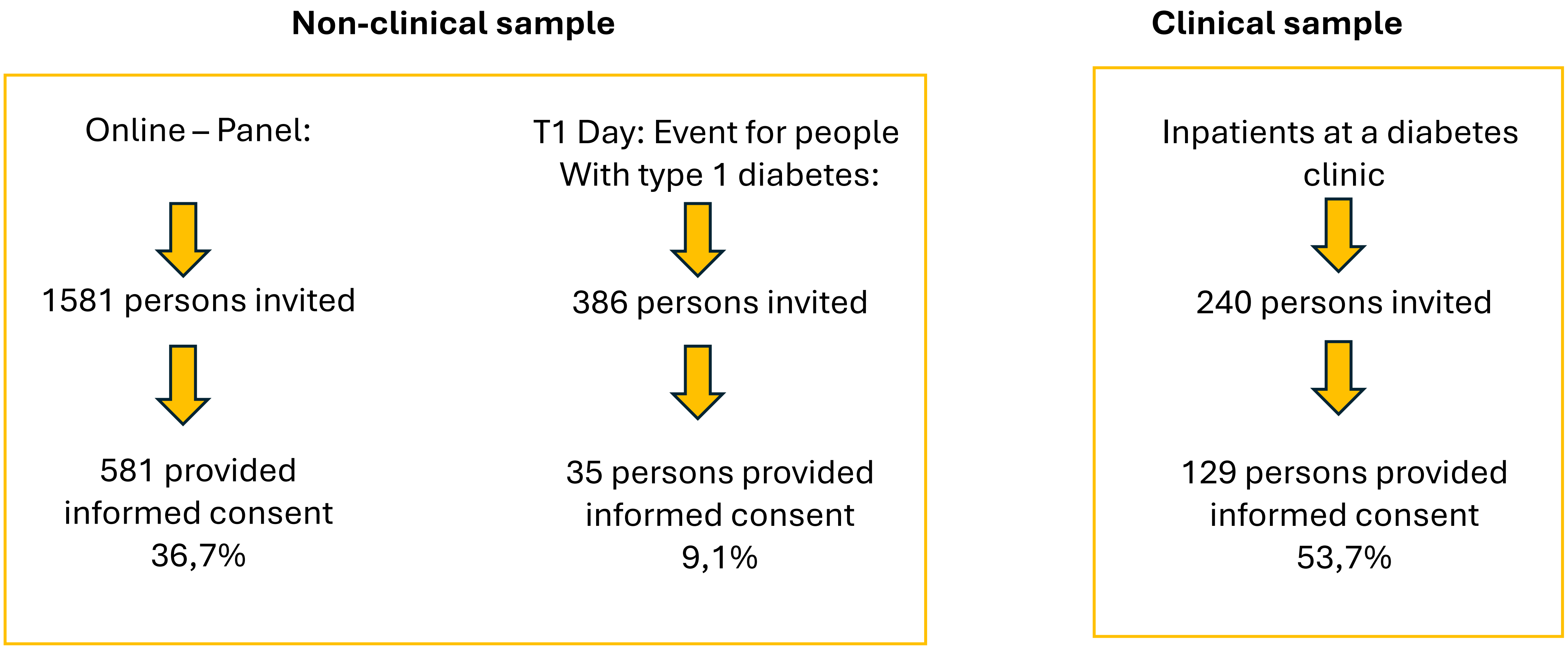

Supplementary Figure 1: Study flow chart

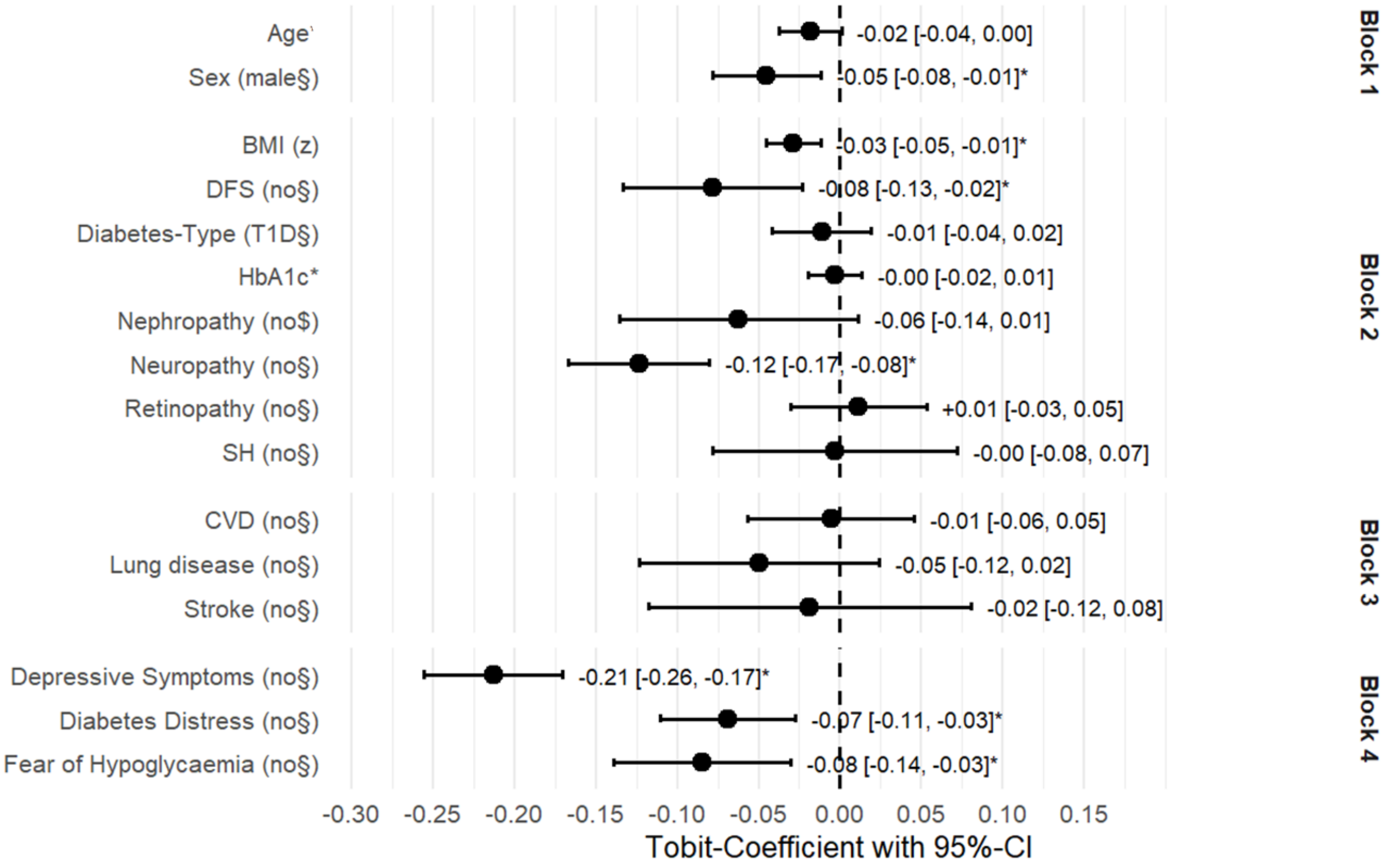

Supplementary Figure 2: Results of the complete multivariate Tobit regression analysis between EQ-5D_L5_utility_ index (dependent variable) and potential statistical predictors (independent variable) with p-values < 0.15 in univariate analysis.

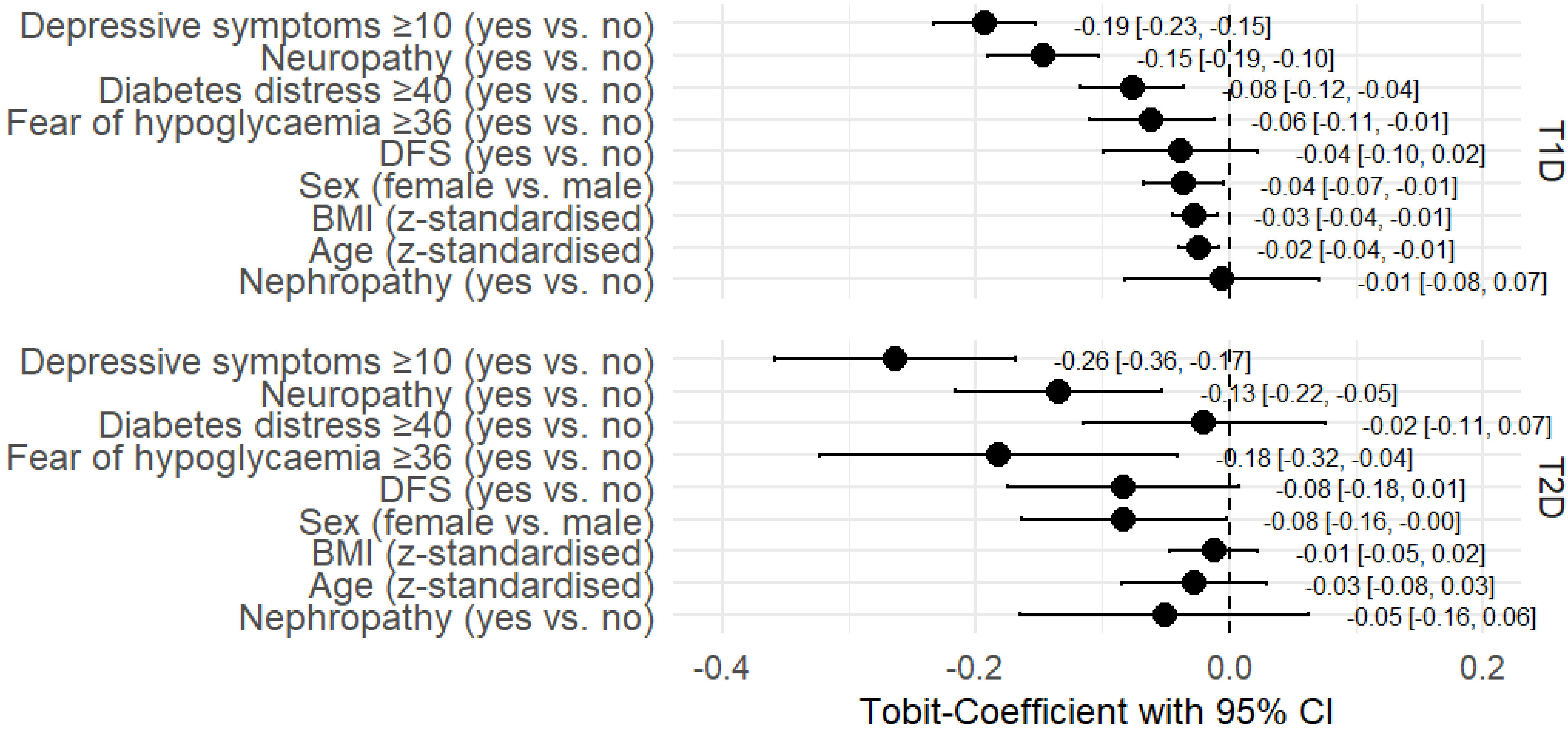

Supplementary Figure 3: Results of a sensitivity/robustness analysis stratified by diabetes type

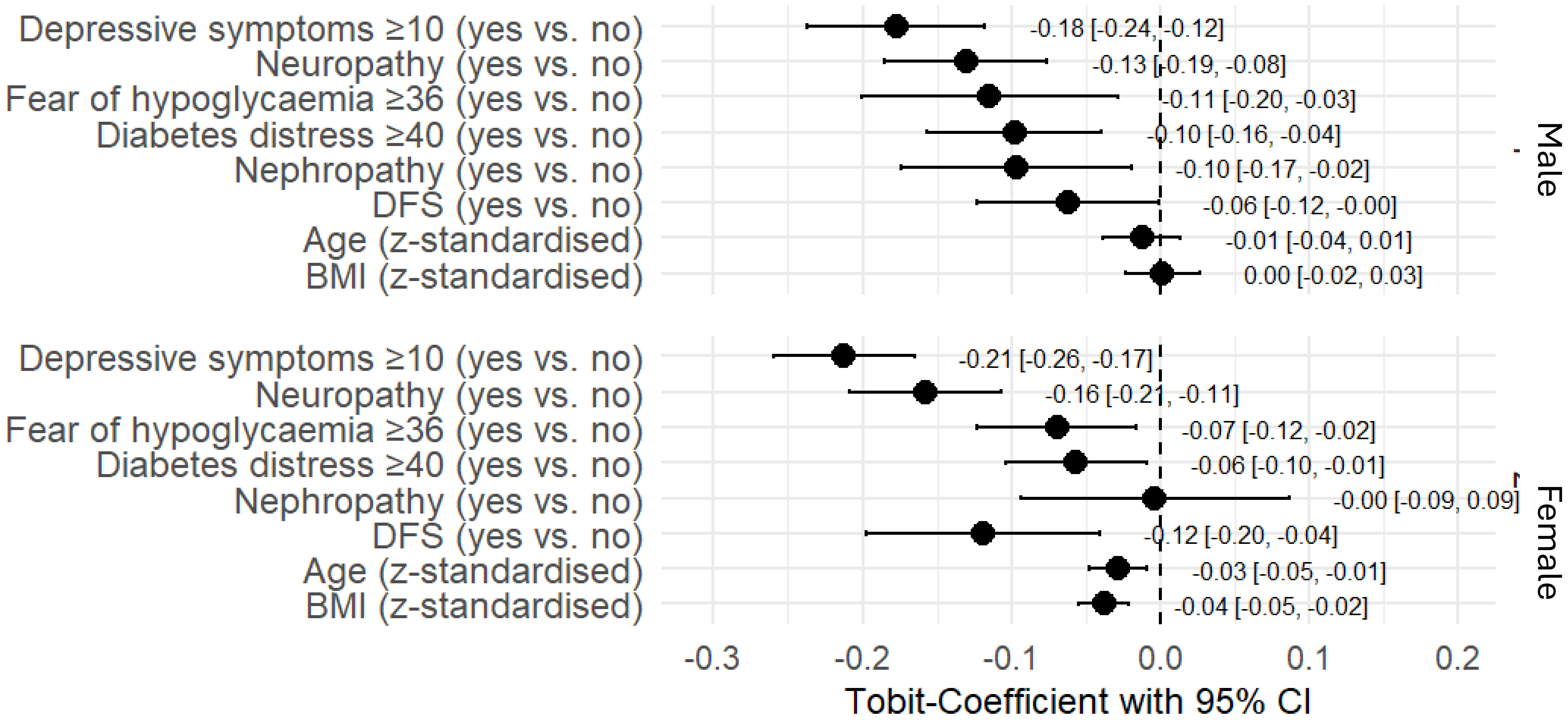

Supplementary Figure 3: Results of a sensitivity/robustness analysis stratified by gender

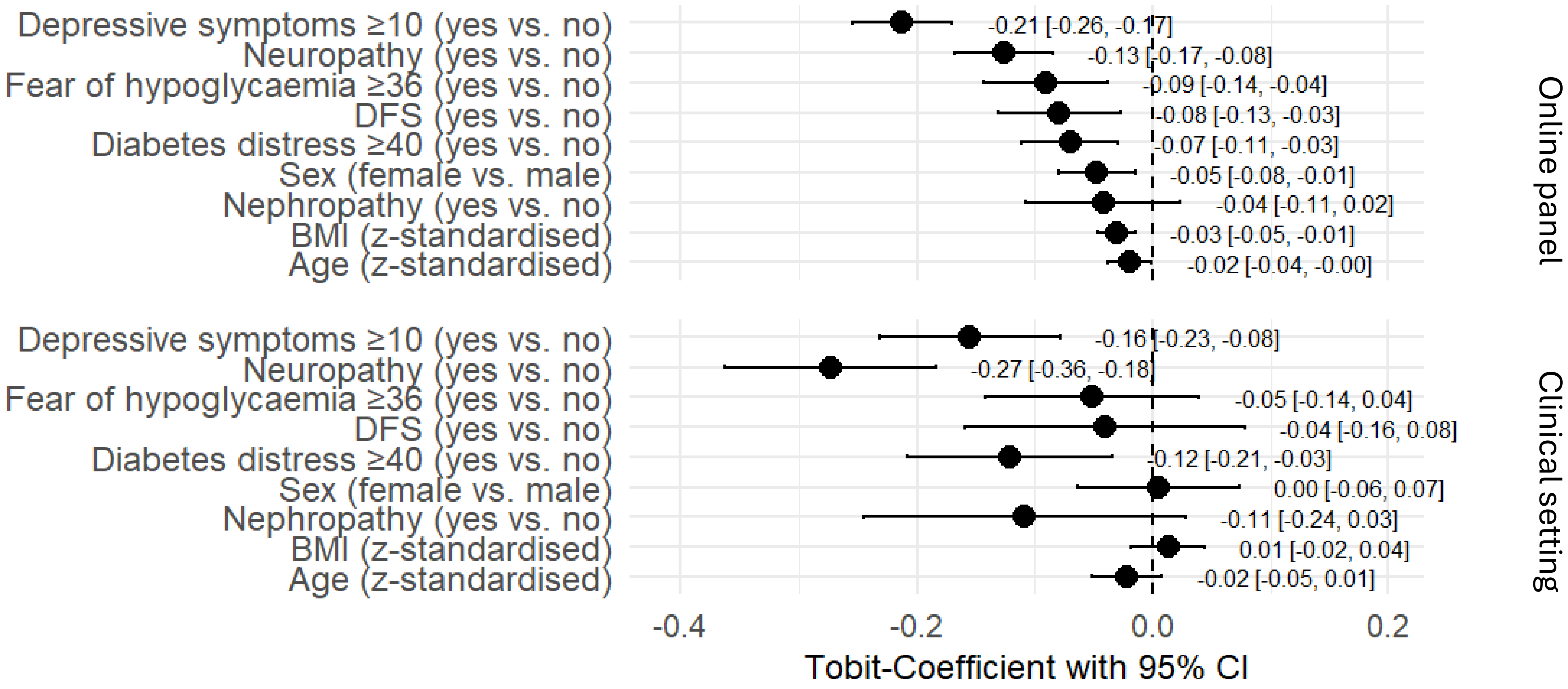

Supplementary Figure 5: Results of a sensitivity/robustness analysis stratified by recrutment-setting

Table S1: Omnibus interaction tests: ALL predictors × subgroup

Subgroup	LR	df	p	N	K
Gender	17.51	8	0.02518	671	8
Diabetes_type	8.12	8	0.4218	637	8
clinical	13.77	8	0.08793	671	8

Table S2: Omnibus interaction tests per interaction subgoups (gender, diabetes-type and recruitment setting) x predictor (Wald p-values)

Interaction	PredictorBase	SubgroupLevel	Estimate	StdError	p
Depression:Gender	Depression	2	-0.03481	0.03871	0.3685
Neuropathy:Gender	Neuropathy	2	-0.02734	0.0379	0.4707
Diabetes Distress:Gender	Diabetes Distress	2	0.04115	0.03842	0.2842
BMI:Gender	BMI	2	-0.03908	0.01544	0.01139
Fear of Hypoglycaemia:Gender	Fear of Hypoglycaemia	2	0.04507	0.0517	0.3833
Diabetic foot syndrome:Gender	Diabetic foot syndrome	2	-0.05673	0.05063	0.2625
Age:Gender	Age	2	-0.01564	0.01656	0.3449
Nephropathy: Gender	Nephropathy	2	0.0933	0.06058	0.1235
Depressen:Diabetes_type	Depression	T2D	-0.06889	0.04604	0.1346
Neuropathy:Diabetes_type	Neuropathy	T2D	0.0205	0.04243	0.629
Diabetes Distress:Diabetes_type	Diabetes Distress	T2D	0.06095	0.04636	0.1886
BMI:Diabetes_type	BMI	T2D	0.01208	0.01753	0.4908
Fear of Hypoglycaemia:Diabetes_type	Fear of Hypoglycaemia	T2D	-0.129	0.0656	0.04921
Diabetic foot syndrome:Diabetes_type	Diabetic foot syndrome	T2D	-0.03423	0.05094	0.5016
Age:Diabetes_type	Age	T2D	0.001625	0.02572	0.9496
Nephropathy:Diabetes_type	Nephropathy	T2D	-0.04218	0.06443	0.5127
Depression:clinical	Depression	1	0.0612	0.05011	0.222
Neuropathy:clinical	Neuropathy	1	-0.1538	0.05799	0.00799[illegible]
Diabetes Distress:clinical	Diabetes Distress	1	-0.05485	0.05658	0.3323
BMI:clinical	BMI	1	0.04273	0.02026	0.03494
Fear of hypoglycaemia:clinical	Fear of Hypoglycaemia	1	0.0452	0.06051	0.4551
Diabetic foot syndrome:clinical	Diabetic foot syndrome	1	0.03341	0.07668	0.6631
Age:clinical	Age	1	-0.007634	0.01955	0.6961
Nephropathy:clinical	Nephropathy	1	-0.08539	0.08833	0.3337
